# Huntington’s Disease Mouse Models Online: High-Resolution MRI Images with Stereotaxic Templates for Computational Neuroanatomy

**DOI:** 10.1371/journal.pone.0053361

**Published:** 2012-12-31

**Authors:** Stephen J. Sawiak, Nigel I. Wood, T. Adrian Carpenter, A. Jennifer Morton

**Affiliations:** 1 Wolfson Brain Imaging Centre, Department of Clinical Neuroscience, University of Cambridge, Cambridge, United Kingdom; 2 Behavioural and Clinical Neuroscience Institute, University of Cambridge, Cambridge, United Kingdom; 3 Department of Pharmacology, University of Cambridge, Cambridge, United Kingdom; King's College London, United Kingdom

## Abstract

Magnetic resonance imaging (MRI) has proved to be an ideal modality for non-destructive and highly detailed assessment of structural morphology in biological tissues. Here we used MRI to make a dataset of *ex vivo* brains from two different rodent models of Huntington’s disease (HD), the R6/2 line and the YAC 128 mouse. We are making the whole dataset (399 transgenic HD and wildtype (WT) brains, from mice aged 9–80 weeks) publicly available. These data will be useful, not only to investigators interested in the study of HD, but also to researchers of computational neuroanatomy who may not have access to such large datasets from mouse models. Here we demonstrate a number of uses of such data, for example to produce maps of grey and white matter and cortical thickness. As an example of how the library might provide insights in mouse models of HD, we calculated whole brain grey matter volumes across different age groups with different numbers of cytosine-adenine-guanine (CAG) repeats in a fragment of the gene responsible for HD in humans. (The R6/2 dataset was obtained from an allelic series of R6/2 mice carrying a range of CAG repeat lengths between 109 and 464.) This analysis revealed different trajectories for each fragment length. In particular there was a gradient of decreasing pathology with longer CAG repeat lengths, reflecting our previous findings with behavioural and histological studies. There will be no constraints placed on the use of the datasets included here. The original data will be easily and permanently accessible via the University of Cambridge data repository (http://www.dspace.cam.ac.uk/handle/1810/243361).

## Introduction

Huntington’s disease (HD) is an inherited, fatal, autosomal dominant disease characterised by movement disorder and psychiatric symptoms [1]. The disease results from a repeated polyglutamine expansion in the coding region of the *HTT* gene. There is no cure for HD and there are few effective treatments. A large number of transgenic mouse models of HD have been developed to study pathogenesis and investigate potential treatments; the most widely used of these is the R6/2 mouse model that typically carries a CAG repeat length of 110–250 [2]. This fragment mouse model shows a progressive phenotype that recapitulates a number of features of the human condition including motor disturbances, stereotypic movements, weight loss and cognitive abnormalities [3]–[5]. As in the human disease, CAG repeat lengths appear to be associated with disease onset and severity [6]. However, mice with extreme repeat lengths in this model present with a disease having a delayed phenotype. This delay in the onset and reduction in the severity of symptoms, in parallel with neurodegenerative changes, provides a model with the potential to elucidate more of the underlying pathogenesis [7]. In addition to the R6/2 model, a number of other models have been made that were aimed at recapitulating better the genetics of HD. These include full length knock-in models [8], [9], a yeast artificial chromosome (YAC128) model [6], and a bacterial artificial chromosome (BAC) model [10].

Historically, magnetic resonance imaging (MRI) findings for individual patients were diagnostic only in later stages of HD, for example where caudate atrophy contributed to the characteristically large ventricles seen [11]. More recently, analytical methodologies, such as tensor-based morphometry (TBM), have been used to show progressive structural changes in presymptomatic HD patients [12]. Neuroimaging studies based on voxel-based morphometry (VBM) are also used widely to investigate developing pathology in humans and assess prospective treatments [13]–[18]. These automated methods for characterising structural differences or changes in the living brain have also been used in mouse models to show that many pathological features are shared between the mouse models and humans with the disease [19]–[22].

Here we describe a large dataset of MR images of mice used in models of HD that includes transgenic R6/2 lines of various CAG expansion lengths, yeast artificial chromosome (YAC128) [23] and wildtype (WT) mice. In addition, we include the MRI data sets from a colony of complexin 1 knockout (Cplx1 KO) mice that showed subtle morphological abnormalities detectable with MRI [24] that reflect behavioural abnormalities seen in the mice [25].

We have recently published two studies using a small subset of these brains (n = 88), where we used manual volumetry and VBM to characterise differences between WT and R6/2 mice [20], [21]. A novel aspect of that work was the use of segmented grey matter (GM) and white matter (WM) in the mouse brain, an approach that is not widely used outside the human brain, despite its success in patients and healthy controls. The most common alternative approach to automated analysis involves ignoring the images once they have been registered to a common atlas and instead performing statistical tests on the registration parameters (tensor- or deformation-based morphometry, see e.g. [26], [27]). Retaining some image intensity information in the form of GM maps allows greater scope for chemical changes that are not associated with volume changes to be observed. Using measures of shape change to compare brains, such as the Jacobian determinant of transformation fields, will reveal only microstructural changes when these cause the registration model to geometrically warp the brain to ‘correct’ the differences in signal as a geometric change rather than one in chemical environment. This is particularly relevant here, as we have shown that not only are there size differences in key brain regions of the R6/2 mouse, but also signal intensity changes [21].

We are releasing these datasets to the neuroscience community to facilitate research into structural differences seen in mice and to provide common datasets that can be used for advancing methodological techniques of automated assessment of structural phenotypes. We are also releasing online the structural data, segmented GM and WM tissue maps for each brain, as well as population-average templates that can be used for VBM investigations [27]. To show how these data might be used, here we present sample results from automated whole brain volume assessment across ages in WT mice and sub-strains of R6/2 mice with differing cytosine-adenine-guanine (CAG) repeat lengths, as well as brains from YAC128 and complexin 1 knockout (Cplx1 KO) mice. In addition, we present maps showing the cortical thickness variation between strains. All of the datasets are available via DSpace, the Institutional Repository of the University of Cambridge (permanent link: http://www.dspace.cam.ac.uk/handle/1810/243361). Once lodged, files will remain accessible indefinitely. As well as the images, metadata describing the age, sex, and other relevant model details (e.g. for R6/2 mice the CAG expansion length) will be included. In addition to the image data, we have provided templates and open-source extension software (SPMMouse; http://www.spmmouse.com) permitting the analysis of these and other animal brains in the popular SPM package that is widely used throughout the neuroimaging community (Wellcome Trust Centre for Neuroimaging, University College London, UK).

We are continuing to acquire images, in particular from longitudinal scans acquired *in vivo*. These will be added to our open-access library *ad hoc* as they become available. All of the datasets presented here were acquired *post mortem* either as an intact head or following skull extraction as described in the methods section.

## Materials and Methods

### Mice

All experiments were conducted in accordance with the UK Animals (Scientific Procedures) Act 1986, and with the approval of the University of Cambridge Licence Review Committee.

Mice were taken from colonies of R6/2, YAC128 and Cplx1 KO mice established in the University of Cambridge. Mice were kept from weaning in home cages comprising single sex, single genotype groups of 10. All of the mice lived in an enhanced environment with increased amounts of bedding and nestling materials. Clean cages were provided twice weekly, with grade 8/10-corncob bedding, and finely shredded paper for nesting. Genotyping was performed using PCR from tail snips taken at 3 weeks and CAG repeat lengths were measured by Laragen (Los Angeles, CA,USA). The mice were maintained on a 12 hour light : 12 hour dark cycle with humidity regulated to 55±10% and temperature from 21–23°C. The mice had *ad libitum* access to water (using water bottles with elongated spouts) and dry laboratory food (RM3(E) rodent pellets, Special Diet Services, Witham, UK). In addition, once a day, a mash was prepared by soaking 100g dry food in 230 ml water until the pellets were soft and fully expanded. The mash was placed on the cage floor, improving access to food and water for the mice. This feeding regime has been shown previously to be beneficial for R6/2 mice [28] and so has become standard practice for all of our mouse colonies.

R6/2 mice [2] were maintained by backcrossing them onto CBAxC57Bl/6 F1 female mice. Genotyping and CAG repeat length measurement were carried out by Laragen (Los Angeles, CA, USA). R6/2 mice were divided into groups according to CAG repeat length. (Table 1).

**Table 1:** Number of mice in library by genotype and sex.

		CAG repeat length			Sex	
Mouse strain		Range	Mean	SD	Male	Female
	***n***				***n***	***n***
**WT (in-skull)**	**124**	**−**	**−**	**−**	**62**	**62**
**WT (ex-skull)**	**61**	**−**	**−**	**−**	**40**	**21**
**R6/2 (in-skull)**						
Super short	25	109–114	112	1.4	15	10
Short	8	182–187	185	1.5	3	5
Medium	43	224–299	255	15	13	30
Long	21	305–391	353	29	12	9
Super long	15	459–464	461	2.1	4	11
**R6/2 ex-skull**						
Medium	**62**	230–257	250	3.3	**27**	**35**
**All R6/2**	**174**	109–464			**74**	**100**
**YAC**	**22**				**9**	**13**
**Cplx1 KO**	**18**				**8**	**10**
**All brains**	**399**				**193**	**206**

YAC128 mice [6] were maintained on a C57/Bl6 background and genotyped by Laragen (Los Angeles, CA, USA).

Cplx1 KO mice [29] were bred on a mixed genetic background (129Ola × C57/Bl6) in a colony established in the Department of Pharmacology, University of Cambridge.

Table 1 shows numbers, sex, genotype and CAG repeat lengths for all mice used in this study. Table 2 shows the breakdown by genotype and age of all mice used in this experiment.

**Table 2:** Number of mice in library by genotype and age.

	Genotype or strain (n)				
Age (weeks)	WT	R6/2	YAC	Cplx1 KO	Total number
9–12	9	10	**−**	**−**	**19**
12–15	71	80	**−**	**−**	**151**
15–18	7	3	**−**	**−**	**10**
18–22	58	73	**−**	**−**	**131**
30–45	3	8	**−**	**5**	**16**
45–55	32	**−**	22		54
55–80	5			13	18
**Total**	**185**	**174**	**22**	**18**	**399**

### Brain Preparation

Mice were deeply anaesthetised with Euthatal, then perfused with 200 ml ice-cold PBS and fixed with 300 ml of ice-cold paraformaldehyde (2% in PBS). In our initial protocol, brains were extracted from the skull and post-fixed overnight in 2% paraformaldehyde and cryoprotected in 30% sucrose in PBS (plus 0.02% sodium azide) for 2 days. We refined this protocol for imaging inside the skull to protect the brain tissues, in particular, the pial surface and olfactory bulbs. The earliest acquisitions made were performed with the skull removed so that a smaller solenoid coil could be used for better image quality. We became concerned, however, that damage to the brain that could occur during extraction (in particular to the cortical surface) could limit our ability to detect subtle differences in these regions. All later acquisitions were therefore scanned with the skull intact. Full details of the preparation of the mice used in the construction of the library are shown in tables 1 and 2.

### Image Acquisition

We followed protocols designed for optimal contrast between grey and white matter. The selected schemes for in-skull and out-of-skull imaging are described below.

#### In-skull imaging

Brains were scanned using a 4.7T Bruker PharmaScan system using a 20cm birdcage coil for transmission and reception. A rapid acquisition with relaxation enhancement (RARE) sequence was used (repetition time (TR)/echo time (TE) 2000/30 ms, echo train length (ETL) 8, number of excitations (NEX) 2) total scan time 3.5 hours per brain. The imaging matrix was 256×192×128 over a field of view 17.9×13.4×9.0 mm^3^ yielding an isotropic resolution of 70µm^3^.

#### Ex-skull imaging

Brains were scanned in Fluorinert FC-77 (3 M), a proton-free susceptibility-matching fluid, using a 1T Bruker system (Bruker Corporation, Ettlingen, Germany) with a 13 mm solenoid coil for signal transmission and reception. A RARE sequence was used for acquisition, optimised for contrast between GM and WM (TR/TE 2000/50.5 ms, ETL 4, NEX 4), and giving a total scan time 13.5 hours per brain. The field of view and matrix parameters were the same as for the in-skull protocol.

### Post-processing

All images were reconstructed and stored in NIFTI format (Neuroimaging Informatics Technology Initiative: http://nifti.nimh.nih.gov/). Header matrices were updated for rigid alignment approximately to Paxinos coordinates [30] by manual alignment to the tissue probability maps (TPMs) created in our previous VBM study [20]. Specifically, the NIFTI headers of the MRI files contain a coordinate transformation matrix that maps voxel coordinates to approximate bregma in millimetres with a rigid transformation. This was done by visual comparison of landmarks between the atlases to align the origin of the mapped coordinate system to bregma points in the Paxinos atlas. Storing this matrix in the header (without modifying the original data) avoids excessive interpolation which could degrade image quality for subsequent processing steps.

### Canonical Tissue Class Maps

SPM5 (Wellcome Trust Centre for Neuroimaging, UK) with the SPMMouse toolbox (http://www.wbic.cam.ac.uk/~sjs80/spmmouse.html) was used for affine registration followed by non-linear registration, radiofrequency inhomogeneity correction and segmentation based on prior knowledge using the unified segmentation model [31]. The priors used were those of our previous VBM study. The resulting modulated maps of GM, WM and CSF were averaged to produce new probability maps and the process was repeated. These processing steps were performed separately for in- and out-of-skull brains due to the differences in shape between them. For each brain, we provide the native space image, the native space segmented GM and WM images and the modulated normalised images in template space.

### Voxel-based Cortical Thickness Maps

**Figure 1:**
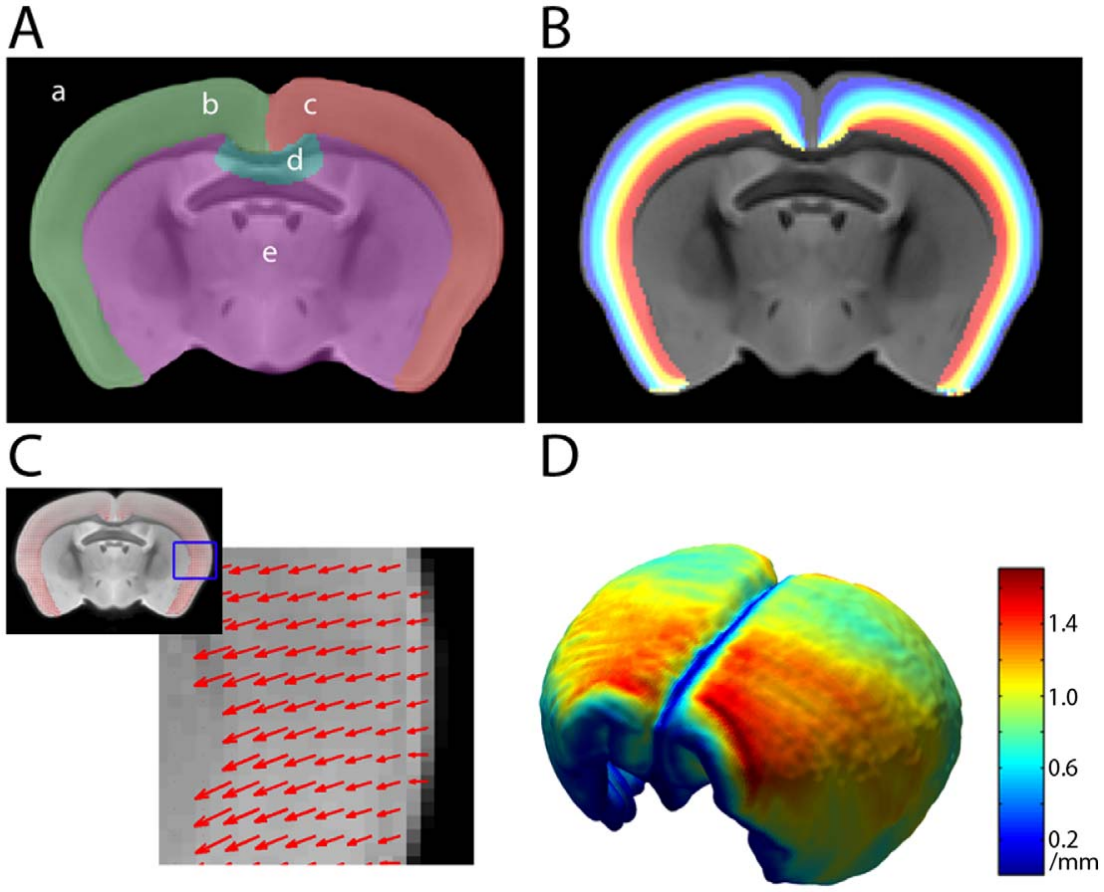
Steps involved in the evaluation of cortical thickness measures. A: regions of potentials providing boundary conditions for solving Laplace’s equation (external region (a), cortical regions (b-c), resistive region (d) and internal region (e)) B: the solved potential in the cortex; C: integration of field lengths to produce D: the thickness map for the cortex shown as a 3D map.

Maps of cortical thickness for each brain were prepared by first delineating the cortical hemispheres on the atlas image. Thicknesses were evaluated by solving Laplace’s equation with potential boundaries on the internal and external cortical surfaces with a ‘resistive’ region for part of the medial cortical boundary following Lerch et al. [19] as illustrated in Figure 1. The cortical regions were transformed via non-linear registration to the native space of each image. In this space the equation was solved to calculate the potential. At each voxel located in the cortex, integration is done in rising and falling directions to reach the inner and outer cortical surfaces, respectively. The sum of these integrals then gives the cortical thickness measure at that point. These maps were then transformed back to the common stereotactic space.

Methods for performing similar calculations have been used in a number of analyses to date where comparisons have been made to histological and manual measurements (e.g. [32], [33]). In illustration here, a single brain from the library of data here was the subject of detailed measurements in two sections in a three-way comparison of 25 areas of cortex between a manual histological measurement, a manual measurement based on the native-space MR image and the calculated cortical thickness map. Details of the preparation for histology followed our previous protocol [24] and manual measurements were made by a single reviewer on homologous cortical features based on the nearest corresponding points on the MRI slices and histology. The automated measurements corresponding to each of these were given by interpolating the start and end points of the lines drawn to measure the MRI slices.

### Accessing the Data

Datasets can be downloaded from http://dspace.cam.ac.uk/handle/1810/243361. Nifti files can be visualized in every modern medical imaging package (e.g. freely-available ImageJ, US National Institutes of Health, Bethesda http://rsbweb.nih.gov/ij/). Structural analysis of GM/WM can be performed in SPM with our SPMMouse plugin both of which can be freely downloaded from the SPM website (http://fil.ion.ucl.ac.uk; Wellcome Trust Centre for Neuroimaging, University College London, UK).

## Results

**Figure 2:**
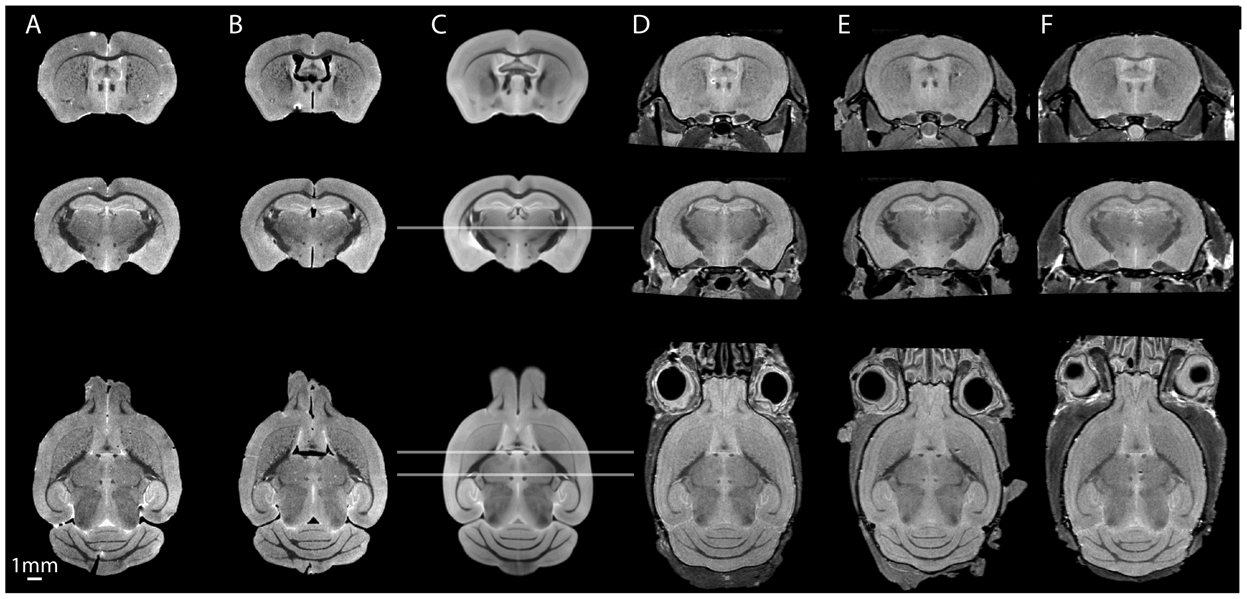
Illustrative examples of images from the library. Columns A-B: WT and R6/2 (CAG250) brains. Column C: atlas image for comparison (see text for details). Columns D-F: in-skull images of WT, R6/2 (CAG250) and YAC128 brains. Horizontal lines in C indicate coronal section positions (bregma −0.8 mm, −1.5 mm, the horizontal slice is taken at bregma 2.8 mm). Note the damaged areas of the ex-skull brains, for example at the olfactory bulbs, and the cerebellar tearing.

Exemplar brain images are shown in Figure 2 for both in-skull and out-of-skull brains. These particular examples show 18 week-old out-of-skull images from WT (Figure 2, column A) and R6/2 CAG250 (Figure 2, column B). Figure 2 column C shows our atlas image constructed from out-of-skull brains. In-skull images are shown from 22 week old WT and R6/2 CAG250 mice (Figure 2 columns D and E, respectively), and from a 45 week old YAC128 mouse (Figure 2, column F).

**Figure 3:**
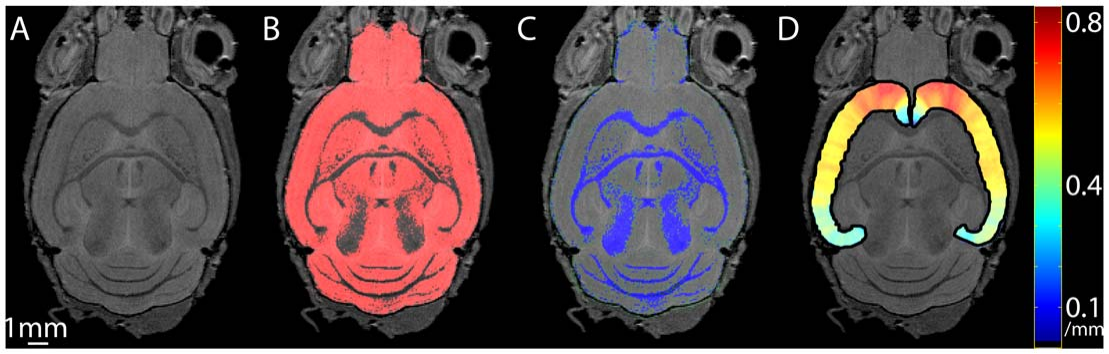
Native space images from the library for a single brain. Raw data from the scanner (A), grey matter segmented map (B, shown in red), white matter segmented map (C, shown in green) and voxel-based cortical thickness map (D).

Examples of the processed data incorporated into our mouse library are shown in Figure 3. For illustration, images from raw data (Figure 3A), GM segmentation (Figure 3B), WM segmentation (Figure 3C) and cortical thickness maps (Figure 3D) are shown.

**Figure 4:**
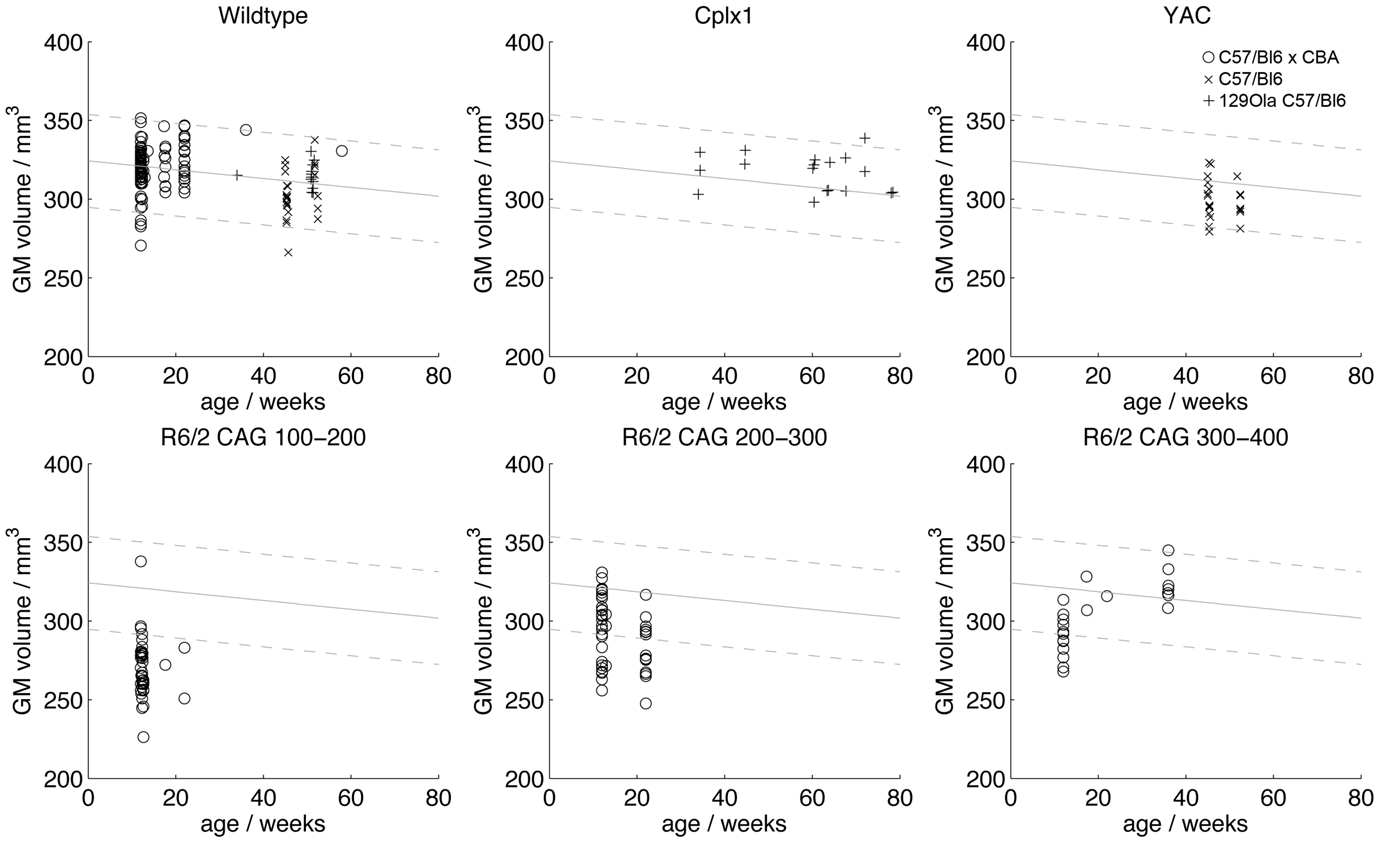
GM volumes for brains from the library. A linear fit to the WT brains (solid line) showing two standard deviations above and below (dashed lines) is repeated on each graph to aid comparison. For the R6/2 lines these data clearly show different age trajectories for different CAG repeats.

The segmented tissue maps can be used readily to calculate gross morphological metrics from each brain. For example Figure 4 shows total GM volume for WT brains, with R6/2 (CAG100–200), (CAG200–300) and (CAG300–400) mice for comparison. If deviation from WT GM volume is considered to be a crude metric of disease burden, it can be seen that mice with longer CAG repeats show a less severe form of disease for comparable age points in the R6/2 lines. These data are consistent with our histopathological findings and behavioural/cognitive data [7]. For completeness, graphs for YAC and Cplx1 KO brains are also shown. To aid comparison, the least-squares line for the WT series has been replicated on each panel, together with dashed lines above and below both shown at twice the standard deviation of the residuals of WT brains to the linear fit.

**Figure 5:**
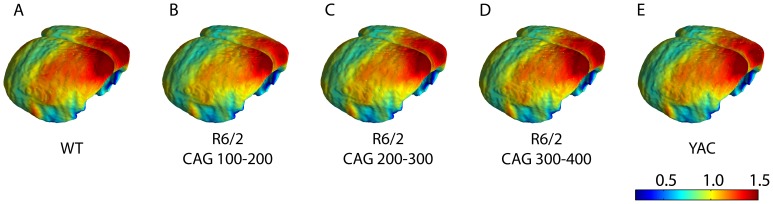
Reconstructed cortex images showing cortical thickness for brains from the library, scale bar in mm.

As an example of possible uses of the datasets, cortical thickness maps, reconstructed as 3D brain models in Figure 5, show thinning in R6/2 with shorter repeat lengths particularly in sensorimotor cortex and frontal areas. Taking a small (four voxel) region centred in sensorimotor cortex (S1; bregma coordinates (3.20 mm left-right, 0.60 mm anterior-posterior, 2.5 mm inferior-superior) shows significant decreases of 3.5% and 4.0% in R6/2 (CAG 100–200) and R6/2 (CAG 200–300) series mice with p-values of 0.002 and 0.004. No significant decreases were seen at this location for R6/2 (CAG 300–400) or the YAC mice (p>0.05). These p-values were calculated with two-tailed Student’s *t*-tests using Matlab 7. These data could be exploited in other ways, for example in performing voxel-based cortical thickness comparisons [34] between mouse models or within-group comparisons based on covariates such as age or sex.

**Figure 6:**
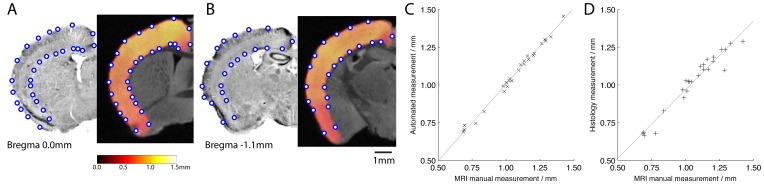
Comparisons between histological cortical thickness measures with automated and manual MRI measurements. Histological and MRI manual measurement points made on the same brain at bregma 0.0 mm (A) and bregma −1.1 mm (B). Graphs showing the relationship between manual MRI measurements and manual histological measurements (C) and manual MRI measurements with the automated cortical thickness measures at the same points (D).

Figure 6 shows the comparison made between MR images and histology for the closest matching slices between the histological dataset and native-space image for the same brain including in the library. It is clear that some shrinkage has occurred in the histological preparation leading to the histological measurements being systematically lower than the MRI measurements (though with good agreement: linear least-squares regression slope 0.91, R^2^ = 0.94). The agreement between manual MRI-based and automated MRI-based cortical thickness measurements is better (regression slope 1.01, R^2^ = 0.99) than the agreement between manual MRI-based measures and manual histological measures.

## Discussion

We are making our extensive collection of high-resolution MR images of R6/2, YAC128, Cplx1 KO and WT mice publically available to download in permanence for use for any purpose. This will be an invaluable resource for the neuroscience and neuroimaging communities to improve our understanding of the pathogenesis in HD via study of its morphological phenotype.

In addition to standard computational neuroanatomy techniques (VBM etc.) that we have already applied to subsets from this library, in this paper we show some of the other ways in which MRI data can be used to explore differences in transgenic lines used to model HD. For example, a simple extraction of total volumes from the GM maps presented here has shown how whole-brain GM volume shows different age trajectories for the R6/2 lines, adding to mounting evidence of different mechanisms underlying pathology in mice with ‘super-expanded’ CAG expansions. Reconstructed cortical thickness maps are an example of how versatile the images can be and how they are amenable to a wide range of different analytical treatments with high power due to the large number of subjects available. We have shown that the values of cortical thickness obtained by our algorithms are in good agreement with manual measurements, and that both measures correspond well with those that would obtained by images prepared histologically. Since all of the data are now freely available, it is possible for other users to try alternative algorithms for cortical thickness measurement.

The range of HD models included in the library show a range of CAG repeat lengths. There is a growing body of data from behavioural and gene expression studies suggesting that mice carrying extremely long CAG repeat lengths show a delayed onset of phenotype [7], [35], [36]. The explanation for this delay in onset remains unclear, since the mice still die prematurely of a neurological disease [7]. One possibility is that the protein carrying the very long polyglutamine products of superlong CAG repeat-containing gene fragments cannot enter the nucleus, and therefore cannot form the pathological inclusions that are characteristic pathology of mice with shorter repeats. (The pathology in the superlong CAG repeat mice is slowly developing and typically extranuclear.) Although there is no direct clinical analogue of extremely long somatic CAG repeats in patients, nevertheless very expanded CAG repeats are found in human post mortem brain, due to somatic instability [37]–[39]. Interestingly, the mice with superlong CAG repeats show a more human-like brain pathology from those with shorter CAG repeats [7]. The significance of these findings remains to be established, but it is hoped that identified differences in *htt* accumulation and their relationship to onset and progression of illness will suggest appropriate pathways for therapeutic agents and interventions. The data presented here show that the delays seen in phenotype for longer repeat include changes in the morphological phenotype as seen by MRI. Since one of the major goals of animal models of HD is to study the early pathology and potential interventions, the demonstration of changes in MRI phenotype is important particularly as MRI findings are increasingly used to monitor disease onset in patients [40], [41].

Large datasets better capture background variability and allow more subtle effects to be characterized. It is our intention to add files to the library as we continue to acquire more images from mice with different CAG expansions so that the various patterns of disease seen can be studied in depth. In addition, we plan to add our *in vivo* acquisitions to extend this resource. There is no comparable library of publically-available mouse brain datasets available and we hope that our publication will encourage other investigators to make their data available to provide new opportunities for insight into neurodegenerative disease.

We are committed to open-source software and free access to data and in addition to the online database we will share the algorithms used in this manuscript with other users on request.

## References

[1] Walker FO (2007) Huntington’s disease. Lancet 369: 218–228. 17240289 10.1016/S0140-6736(07)60111-1

[2] Mangiarini L, Sathasivam K, Seller M, Cozens B, Harper A, et al (1996) Exon 1 of the HD gene with an expanded CAG repeat is sufficient to cause a progressive neurological phenotype in transgenic mice. Cell 87: 493–506. 8898202 10.1016/s0092-8674(00)81369-0

[3] Carter RJ, Lione LA, Humby T, Mangiarini L, Mahal A, et al (1999) Characterization of progressive motor deficits in mice transgenic for the human Huntington’s disease mutation. J Neurosci 19: 3248–3257. 10191337 10.1523/JNEUROSCI.19-08-03248.1999 PMC6782264

[4] Lione LA, Carter RJ, Hunt MJ, Bates GP, Morton AJ, et al (1999) Selective discrimination learning impairments in mice expressing the human Huntington’s disease mutation. J Neurosci 19: 10428–10437. 10575040 10.1523/JNEUROSCI.19-23-10428.1999 PMC6782405

[5] Luesse HG (2001) Schiefer J, Spruenken A, Puls C, Block F, et al (2001) Evaluation of R6/2 HD transgenic mice for therapeutic studies in Huntington’s disease: behavioral testing and impact of diabetes mellitus. Behav Brain Res 126: 185–195. 11704263 10.1016/s0166-4328(01)00261-3

[6] Slow EJ, van Raamsdonk J, Rogers D, Coleman SH, Graham RK, et al (2003) Selective striatal neuronal loss in a YAC128 mouse model of Huntington disease. Hum Mol Genet 12: 1555–1567. 12812983 10.1093/hmg/ddg169

[7] Morton AJ, Glynn D, Leavens W, Zheng Z, Faull RL, et al (2009) Paradoxical delay in the onset of disease caused by super-long CAG repeat expansions in R6/2 mice. Neurobiol Dis 33: 331–341. 19130884 10.1016/j.nbd.2008.11.015

[8] Lin CH, Tallaksen-Greene S, Chien WM, Cearley JA, Jackson WS, et al (2001) Neurological abnormalities in a knock-in mouse model of Huntington’s disease. Hum Mol Genet 10: 137–144. 11152661 10.1093/hmg/10.2.137

[9] Menalled LB, Sison JD, Dragatsis I, Zeitlin S, Chesselet MF (2003) Time course of early motor and neuropathological anomalies in a knock-in mouse model of Huntington’s disease with 140 CAG repeats. J Comp Neurol 465: 11–26. 12926013 10.1002/cne.10776

[10] Gray M, Shirasaki DI, Cepeda C, Andre VM, Wilburn B, et al (2008) Full-length human mutant huntingtin with a stable polyglutamine repeat can elicit progressive and selective neuropathogenesis in BACHD mice. J Neurosci 28: 6182–6195. 18550760 10.1523/JNEUROSCI.0857-08.2008 PMC2630800

[11] Oliva D, Carella F, Savoiardo M, Strada L, Giovannini P, et al (1993) Clinical and magnetic resonance features of the classic and akinetic-rigid variants of Huntington’s disease. Arch Neurol 50: 17–19. 8418795 10.1001/archneur.1993.00540010013010

[12] Kipps CM, Duggins AJ, Mahant N, Gomes L, Ashburner J, et al (2005) Progression of structural neuropathology in preclinical Huntington’s disease: a tensor based morphometry study. J Neurol Neurosurg Psychiatry 76: 650–655. 15834021 10.1136/jnnp.2004.047993 PMC1739615

[13] Douaud G, Gaura V, Ribeiro MJ, Lethimonnier F, Maroy R, et al (2006) Distribution of grey matter atrophy in Huntington’s disease patients: A combined ROI-based and voxel-based morphometric study. NeuroImage 32: 1562–1575. 16875847 10.1016/j.neuroimage.2006.05.057

[14] Gomez-Anson B, Alegret M, Munoz E, Monte GC, Alayrach E, et al (2009) Prefrontal cortex volume reduction on MRI in preclinical Huntington’s disease relates to visuomotor performance and CAG number. Parkinsonism Relat Disord 15: 213–219. 18632301 10.1016/j.parkreldis.2008.05.010

[15] Hobbs NZ, Henley SM, Ridgway GR, Wild EJ, Barker RA, et al (2010) The progression of regional atrophy in premanifest and early Huntington’s disease: a longitudinal voxel-based morphometry study. J Neurol Neurosurg Psychiatry 81: 756–763. 19955112 10.1136/jnnp.2009.190702

[16] Jech R, Klempir J, Vymazal J, Zidovska J, Klempirova O, et al (2007) Variation of selective gray and white matter atrophy in Huntington’s disease. Mov Disord 22: 1783–1789. 17579363 10.1002/mds.21620

[17] Ruocco HH, Bonilha L, Li LM, Lopes-Cendes I, Cendes F (2008) Longitudinal analysis of regional grey matter loss in Huntington disease: effects of the length of the expanded CAG repeat. J Neurol Neurosurg Psychiatry 79: 130–135. 17615168 10.1136/jnnp.2007.116244

[18] Tabrizi SJ, Scahill RI, Durr A, Roos RA, Leavitt BR, et al (2011) Biological and clinical changes in premanifest and early stage Huntington’s disease in the TRACK-HD study: the 12-month longitudinal analysis. Lancet Neurol 10: 31–42. 21130037 10.1016/S1474-4422(10)70276-3

[19] Lerch JP, Carroll JB, Dorr A, Spring S, Evans AC, et al (2008) Cortical thickness measured from MRI in the YAC128 mouse model of Huntington’s disease. Neuroimage 41: 243–251. 18387826 10.1016/j.neuroimage.2008.02.019

[20] Sawiak SJ, Wood NI, Williams GB, Morton AJ, Carpenter TA (2009) Voxel-based morphometry in the R6/2 transgenic mouse reveals differences between genotypes not seen with manual 2D morphometry. Neurobiol Dis 33: 20–27. 18930824 10.1016/j.nbd.2008.09.016

[21] Sawiak SJ, Wood NI, Williams GB, Morton AJ, Carpenter TA (2009) Use of magnetic resonance imaging for anatomical phenotyping of the R6/2 mouse model of Huntington’s disease. Neurobiol Dis 33: 12–19. 18930823 10.1016/j.nbd.2008.09.017

[22] Shan ZY, Parra C, Ji Q, Ogg RJ, Zhang Y, et al (2006) A digital pediatric brain structure atlas from T1-weighted MR images. Med Image Comput Comput Assist Interv Int Conf Med Image Comput Comput Assist Interv 9: 332–339. 10.1007/11866763_41 17354789

[23] Hodgson JG, Agopyan N, Gutekunst CA, Leavitt BR, LePiane F, et al (1999) A YAC mouse model for Huntington’s disease with full-length mutant huntingtin, cytoplasmic toxicity, and selective striatal neurodegeneration. Neuron 23: 181–192. 10402204 10.1016/s0896-6273(00)80764-3

[24] Kielar C, Sawiak SJ, Navarro Negredo P, Tse DH, Morton AJ (2012) Tensor-based morphometry and stereology reveal brain pathology in the complexin1 knockout mouse. PLoS One 7: e32636. 22393426 10.1371/journal.pone.0032636 PMC3290572

[25] Glynn D, Sizemore RJ, Morton AJ (2007) Early motor development is abnormal in complexin 1 knockout mice. Neurobiol Dis 25: 483–495. 17188502 10.1016/j.nbd.2006.10.011

[26] Ashburner J, Friston KJ (2000) Voxel-based morphometry–the methods. Neuroimage 11: 805–821. 10860804 10.1006/nimg.2000.0582

[27] Ashburner J (2009) Computational anatomy with the SPM software. Magn Reson Imaging 27: 1163–1174. 19249168 10.1016/j.mri.2009.01.006

[28] Carter RJ, Hunt MJ, Morton AJ (2000) Environmental stimulation increases survival in mice transgenic for exon 1 of the Huntington’s disease gene. Mov Disord 15: 925–937. 11009201 10.1002/1531-8257(200009)15:5<925::aid-mds1025>3.0.co;2-z

[29] Reim K, Mansour M, Varoqueaux F, McMahon HT, Sudhof TC, et al (2001) Complexins regulate a late step in Ca2+-dependent neurotransmitter release. Cell 104: 71–81. 11163241 10.1016/s0092-8674(01)00192-1

[30] Paxinos G, Franklin KBJ (2004) The mouse brain in stereotaxic coordinates. Amsterdam; London: Elsevier Academic Press. 1 v. (unpaged) p.

[31] Ashburner J, Friston KJ (2005) Unified segmentation. Neuroimage 26: 839–851. Epub 2005 Apr 2001. 10.1016/j.neuroimage.2005.02.018 15955494

[32] Carroll JB, Lerch JP, Franciosi S, Spreeuw A, Bissada N, et al (2011) Natural history of disease in the YAC128 mouse reveals a discrete signature of pathology in Huntington disease. Neurobiol Dis 43: 257–265. 21458571 10.1016/j.nbd.2011.03.018

[33] Vernon AC, Crum WR, Johansson SM, Modo M (2011) Evolution of extra-nigral damage predicts behavioural deficits in a rat proteasome inhibitor model of Parkinson’s disease. PLoS One 6: e17269. 21364887 10.1371/journal.pone.0017269 PMC3045435

[34] Hutton C, De Vita E, Ashburner J, Deichmann R, Turner R (2008) Voxel-based cortical thickness measurements in MRI. Neuroimage 40: 1701–1710. 18325790 10.1016/j.neuroimage.2008.01.027 PMC2330066

[35] Dragatsis I, Goldowitz D, Del Mar N, Deng YP, Meade CA, et al (2009) CAG repeat lengths>or = 335 attenuate the phenotype in the R6/2 Huntington’s disease transgenic mouse. Neurobiol Dis 33: 315–330. 19027857 10.1016/j.nbd.2008.10.009 PMC4461140

[36] Tang B, Seredenina T, Coppola G, Kuhn A, Geschwind DH, et al (2011) Gene expression profiling of R6/2 transgenic mice with different CAG repeat lengths reveals genes associated with disease onset and progression in Huntington’s disease. Neurobiol Dis 42: 459–467. 21334439 10.1016/j.nbd.2011.02.008 PMC3079804

[37] Kennedy L, Evans E, Chen CM, Craven L, Detloff PJ, et al (2003) Dramatic tissue-specific mutation length increases are an early molecular event in Huntington disease pathogenesis. Hum Mol Genet 12: 3359–3367. 14570710 10.1093/hmg/ddg352

[38] Shelbourne PF, Keller-McGandy C, Bi WL, Yoon SR, Dubeau L, et al (2007) Triplet repeat mutation length gains correlate with cell-type specific vulnerability in Huntington disease brain. Hum Mol Genet 16: 1133–1142. 17409200 10.1093/hmg/ddm054

[39] Swami M, Hendricks AE, Gillis T, Massood T, Mysore J, et al (2009) Somatic expansion of the Huntington’s disease CAG repeat in the brain is associated with an earlier age of disease onset. Hum Mol Genet 18: 3039–3047. 19465745 10.1093/hmg/ddp242 PMC2714728

[40] Tabrizi SJ, Langbehn DR, Leavitt BR, Roos RA, Durr A, et al (2009) Biological and clinical manifestations of Huntington’s disease in the longitudinal TRACK-HD study: cross-sectional analysis of baseline data. Lancet Neurol 8: 791–801. 19646924 10.1016/S1474-4422(09)70170-X PMC3725974

[41] Tabrizi SJ, Reilmann R, Roos RA, Durr A, Leavitt B, et al (2012) Potential endpoints for clinical trials in premanifest and early Huntington’s disease in the TRACK-HD study: analysis of 24 month observational data. Lancet Neurol 11: 42–53. 22137354 10.1016/S1474-4422(11)70263-0

